# Therapeutic Management of Metastatic Thymoma and Thymic Carcinoma

**DOI:** 10.1007/s11912-025-01680-4

**Published:** 2025-05-28

**Authors:** Nicolas Girard

**Affiliations:** 1 Department of Medical Oncology, Institut Curie, Paris, France; 2 Institut du Thorax Curie Montsouris, Institut Mutualiste Montsouris, Paris, France; 3 UVSQ, Paris Saclay, Versailles, France; 4 Institut Curie, 26, rue d’Ulm, Paris, 75005 France; 5 EURACAN, Lyon, France

**Keywords:** Thymoma, Thymic carcinoma, Targeted therapy, Immunotherapy, Chemotherapy

## Abstract

**Purpose of the Review:**

Assess new options and best sequence or combination strategies for the treatment of metastatic thymic epithelial tumors.

**Recent Findings:**

Besides historical cytotoxic chemotherapy regimens, which remain standard-of-care for many patients with thymoma, new options include antiangiogenic agents and immune checkpoint inhibitors (ICIs) in the first-line setting combined with carboplatin and paclitaxel for thymic carcinoma. Antiangiogenic agents are also used in the second-line setting, possibly sequenced or combined with ICIs. With the latter, comprehensive assessment for autoimmune disorders is advised, with subsequent close clinical and biological monitoring. Precision medicine strategies may be implemented with comprehensive genomic profiling and use of targeted agents.

**Summary:**

Multidisciplinary tumor board is key to optimize the treatment pathway for patients with metastatic thymic epithelial tumors, with a need for prospective studies assessing the best combination strategies.

## Introduction

Thymic epithelial tumors (TETs) are rare mediastinal cancers with an incidence of 0.15–0.25 per 100,000 person-year, which are classified according to the World Health Organization (WHO) histopathologic classification, that distinguishes thymomas from thymic carcinomas [1]; thymomas reproduce the architecture of the thymus and are further subdivided into different types (so-called A, AB, B1, B2, and B3) based upon the relative proportion of the non-tumoral lymphocytic component, and the resemblance to normal thymic architecture. Thymic carcinomas are similar to their extra-thymic counterpart, the most frequent subtype being squamous cell carcinoma. Staging of TETs is currently based on the 9th Edition of the AJCC/UICC TNM staging classification [2]. Thymomas may be associated with autoimmune disorders, such as myasthenia gravis, that require proactive management to ensure accurate conduct of the oncological treatment [1, 2].

The management of TETs is a paradigm of cooperation between clinicians, surgeons, and pathologists from establishing the diagnosis to organizing the multimodal therapeutic strategy [3]. Systemic treatment may be delivered in a curative-intent approach, for patients presenting with locally-advanced tumor at time of diagnosis. In such cases, chemotherapy has been used both to reduce the tumor burden to facilitate complete resection of the tumor which is associated with prolonged survival [3]. A peculiar feature of stage IV thymic tumors is that these may still be eligible for curative-intent multimodal treatment, especially in the setting of pleural invasion or oligometastatic presentation [4]; delivery of Hyperthermic IntraTHOracic Chemotherapy (HITHOC) is then considered [5]. Chemotherapy is also a palliative-intent treatment of unresectable, metastatic, and recurrent tumors, which are more frequently carcinomas than thymomas [6]. Several consecutive lines of chemotherapy may be administered when the patient presents with tumor progression.

**Fig. 1:**
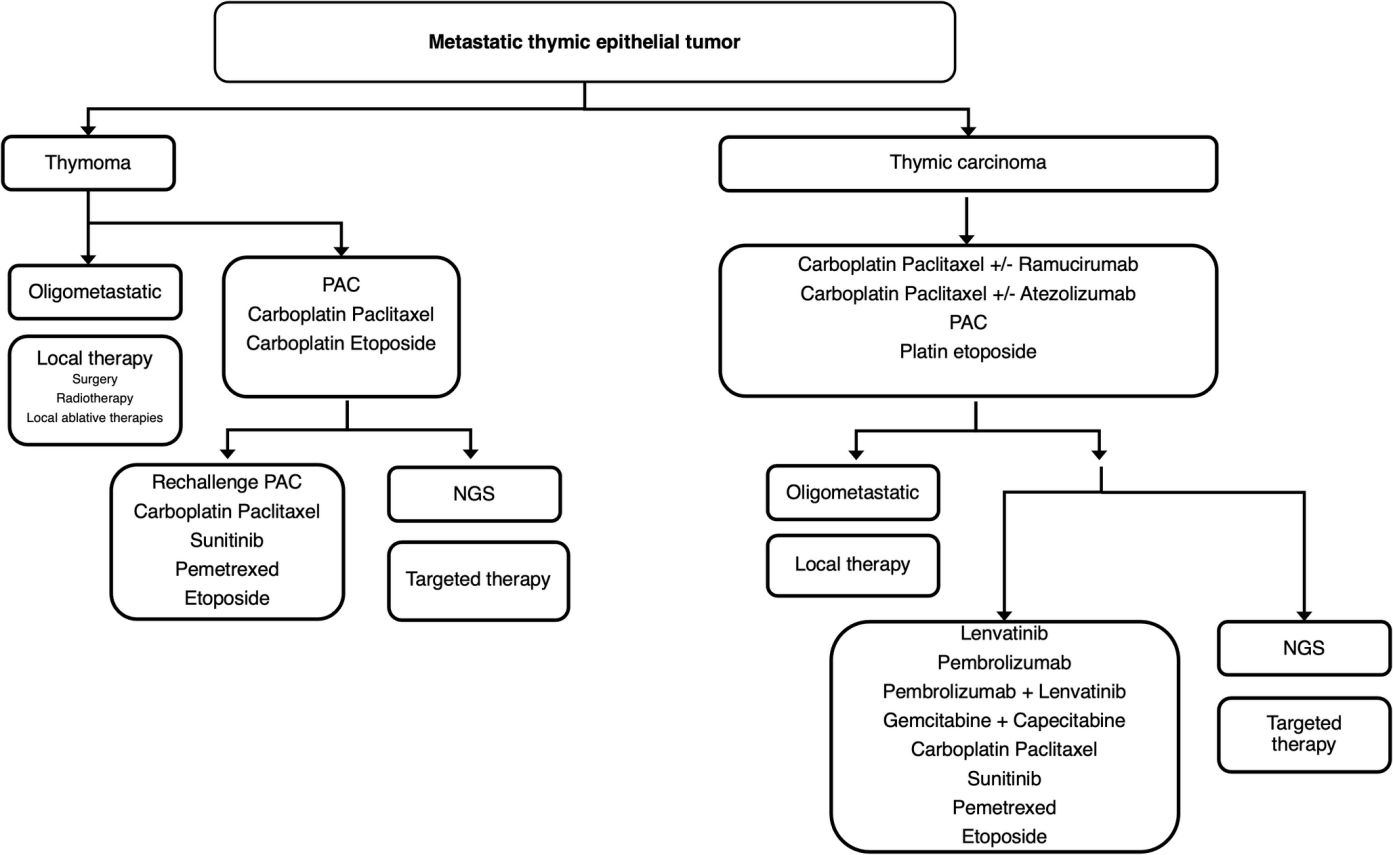
Proposed treatment algorithm for metastatic thymic epithelial tumors

Over the past 5 years, several alternative options for systemic treatment of advanced, refractory thymic tumors have been made available (Fig. 1), including PI3K/MTOR inhibitors, CDK inhibitors, and antiangiogenic agents. Trials were also conducted to assess the efficacy of immune checkpoint inhibitors (ICIs).

## Metastatic and Recurrent Thymic Epithelial Tumors

### Incidence and Risk Factors

Several prognostic factors as a measure of risk of upfront metastatic spread and recurrence after initial treatment for non-metastatic TET have been investigated. Optimal outcome measures in TETs are somehow different from other solid organ tumors because of the indolent behavior of some TETs, particularly thymomas.

The risk of upfront metastatic spread and recurrence after treatment for non-metastatic disease in TETs is ranging from 5 to 20% of cases, and 15-60% of cases, respectively [7, 8]. Type B3 thymoma and thymic carcinoma, which account for 25% of cases at time of diagnosis, are more frequently associated with stage IV disease [9].

As for the risk of recurrence, the recognized strongest predictive factors include histology, stage, and resection status of surgery [10, 11]. A complete resection (R0) has consistently found to be an important favorable factor for the patient survival in all stages. The risk of recurrence ranges from 10 to 30% to 30–80% in thymomas and thymic carcinomas, depending on stage [12]. In thymomas, most recurrences occur inside the pleural cavity, whereas distant recurrences are very rare [13]. Mediastinal recurrences are more frequent in thymic carcinomas, observed in up to 80% of cases along with the follow-up; thymic carcinomas are also frequently presenting with extrathoracic metastases, mostly in the liver and bone [10, 14]. Survival is highly variable, from upfront resected stage I-II thymomas which have virtually no impact on the expected survival of patients, to metastatic thymic carcinoma, with median overall survival of 3 to 4 years [10].

### Imaging

The standard imaging to identify metastatic or recurrent TET is cross-sectional imaging, such as chest computed tomography and/or magnetic resonance imaging, which are also recommended for the follow-up after initial treatment [15, 16]. Imaging should be performed with intravenous contrast injection to help identify local invasion and pleural metastases, and aid in biopsy planning by differentiating viable tissue from cystic/necrotic regions; the time interval between follow-up imaging varies based on histology, stage, and first-intent treatment [3]. 18 F-Fluorodeoxyglucose (18 F-FDG) positron emission tomography (PET)-CT is also recommended to detect otherwise occult metastases overlooked by CT [17].

### Need for Biopsy

Biopsy is obviously needed in patients with upfront metastatic TET, to make the diagnosis before systemic therapies are initiated. For recurrences after surgery, decision for rebiopsy remain to be discussed, and may depend on histology, site of tumor lesions, time since initial management, and potential resectability. When the combined clinical and imaging features are typical for recurrence, treatment can be started without a biopsy. Biopsies may be recommended in case of atypical sites of recurrence, need to have histological confirmation in composite TETs or accurate assessment of biomarkers like by comprehensive genomic profiling.

### Autoimmune Disorders

One-third of patients with thymomas present with autoimmune features, notably myasthenia gravis (MG), which is observed in around 15–20% of the patients and is associated with positive anti-acetylcholine receptor antibodies [18]. Other auto-immune disorders affecting neurological, hematological, dermatological, or endocrine systems may also be observed. Autoimmune conditions can occur before, during, or after TET diagnosis and its treatment.

Clinical and/or laboratory diagnosis of auto-immune disorders should then be evaluated not only at diagnosis but also at the time of TET recurrence. Equally, recurrence of TET should be considered in patients who develop newautoimmune conditions or opportunistic infections. Still, these autoimmune disorders are not paraneoplastic disease as these do not follow the actual history of the tumor [19].

In patients with auto-immune disorders, most frequent treatment are corticosteroids or standard oral immunosuppressive agents, and more recently, for MG, anti-neonatal Fc receptor blockers, anti-complement protein C5 or C5 inhibitors. These may impact the oncological treatment of metastatic TETs.

Another crucial point is that patients with clinical MG, or even isolated positive anti-acetylcholine receptor antibodies, receive accurate education about the risks of myasthenic crisis in specific situations such as physical stress, infections, noncompliance with immunotherapy or the administration of certain drugs; live vaccines are contra-indicated after thymectomy for TETs.

## Local Therapies for Metastatic Thymic Epithelial Tumors

As for the first-intent treatment of early-stage or locally-advanced TETs, the treatment strategy for metastatic TET is primarily based on whether complete resection, given its strong prognostic value, may be achievable. As 30% of patients with thymoma present with oligometastatic or oligorecurrent disease, local therapies are then considered, including surgery and possibly radiotherapy.

### Surgery

As discussed above, stage IVA thymomas, even though metastatic, may be resectable upfront or after primary chemotherapy [20, 21]. This may similarly applies to stage IVB, oligometastatic disease, especially for thymoma. For patients with oligorecurrences, especially in the pleura, surgical resection is the standard treatment for operable patients [22, 23], possibly associated with HITHOC in thymomas [5, 24].

HITHOC is performed under general anesthesia and in conjunction with pleurectomy. Inflows and outflow cannulas are placed within the pleural space and connected to a perfusion pump [25]. A highly concentrated dose of chemotherapeutic agent (cisplatin, less frequently anthracyclines and mitomycin) is infused for 60–90 min at a temperature of 41–43 °C. Although no randomized trial has ever been conducted to assess the value of HITHOC, single arm trials tend to report satisfactory outcomes in term of survival (5-year overall survival (OS) of 70% or more) and recurrence/progression-free-survival (higher than 40% at 5 years) [25]. Significantly better outcomes are reported in thymoma vs. thymic carcinoma. A question is whether HITHOC benefit is better upfront in stage IVA disease or at second- or subsequent recurrence.

### Radiotherapy

Besides palliative radiotherapy to threatening or symptomatic metastases, in the bone or the brain, stereotactic ablative radiotherapy (SABR) is being increasingly proposed for oligometastatic or oligorecurrent TETs, particularly those located in the pleural cavity [26]. A prospective study of SABR for TETs demonstrated response rates of 97% and local control of 81% with minimal toxicities [27].

### Interventional Radiology Procedures

Reports of the use of percutaneous radiofrequency or cryotherapy have been published in the setting of pleural implants non amenable to surgical resection because of poor general condition, or after multiple surgeries or radiotherapy have been performed [28].

## Systemic Therapies

### First-Line Regimens

For metastatic or recurrent TETs not amenable to local treatment, cytotoxic chemotherapy is the first-line standard-of-care (Table 1). The most recommended regimen is cisplatin, doxorubicin and cyclophosphamide (CAP) [6, 29, 30], particularly for thymoma with higher response rates as compared to non-adriamycin-based regimens [31]. Carboplatin and paclitaxel is the preferred chemotherapy option for thymic carcinoma, given its significant activity alongside a manageable safety profile, and similar response rates to adriamycin-based chemotherapy [32]. Up to 4–6 cycles are delivered. Platin and etoposide are less used [33]. The expected response rates are approximately 40% for thymoma and 20% for thymic carcinoma [6, 29, 30, 31, 32, 33].

**Table 1:** Preferred chemotherapy regimens for thymic epithelial tumors

Regimen	Agents	Doses	Schedule
CAP [29]	Cisplatin	50 mg/m^2^	Every 3 weeks
	Doxorubicin	50 mg/m^2^	Every 3 weeks
	Cyclophosphamide	500 mg/m^2^	Every 3 weeks
PE [33]	Cisplatin	60 mg/m^2^	Every 3 weeks
	Etoposide	120 mg/m^2^	Days 1–3 every 3 weeks
Carbo-Px [32]	Carboplatin	AUC5-6	Every 3 weeks
	Paclitaxel	200 mg/m^2^	Every 3 weeks
Cape-Gem [38]	Capecitabine	650 mg/m^2^ BID	Days 1–14 every 3 weeks
	Gemcitabine	1000 mg/m^2^	Days 1,8 every 3 weeks
Pemetrexed [39]	Pemetrexed	500 mg/m^2^	Every 3 weeks
Oral etoposide [41]	Etoposide	25 mg TID	Days 1–21 every 4 weeks

Table legend: AUC = area under the curve; BID = *bis in die*; TID = *tris in die*

More recently for thymic carcinomas, combination regimens with antiangiogenic agents or ICIs have been evaluated in phase II trials (Table 2) [34, 35]. The single-arm phase II RELEVENT trial assessed the combination of carboplatin, paclitaxel and ramucirumab reporting on response rate of 80%, median progression-free survival (PFS) of 18.1 months, and median OS of 43.8 months [34]; these figures are higher than that historically reported, closer to 8–12 and 24–36 months, respectively [6, 29, 30, 31, 32, 33]. Of note, these results have not been completely confirmed in the randomized SWOG1701 trial comparing carboplatin and paclitaxel with or without ramucirumab. Ramucirumab arm increased the response rate (80% vs. 40% in chemotherapy arm) but median PFS was similar in both arms (8 months and 7 months, with and without ramucirumab, respectively) [36]. Despite these controversial data, the combination of platinum-based chemotherapy and antiangiogenic agent may represent a new option in the first-line treatment of thymic carcinoma. The second trial of interest in this setting is the MARBLE study, conducted in thymic carcinomas and type B3 thymomas, with the combination of carboplatin paclitaxel and atezolizumab, which reported an overall response rate (ORR) of 56% and a median PFS of 9.6 months, with a high grade ≥ 3 immune-related adverse events (irAEs) in 17% of patients [35]. Here the intent may actually focus on long-term survival, which is not yet mature. The risks of irAEs in thymic carcinoma (see below) are also a challenge in the first-line setting. Ultimately, a trial in Japan is assessing the feasibility of chemotherapy plus lenvatinib and pembrolizumab [37].

**Table 2:** Key outcomes in metastatic type B3 thymoma and thymic carcinoma with first-line chemotherapy and second-line non-chemotherapy agents

	First-line			Second-line and beyond					
Population	B3 thymoma and thymic carcinoma			All histologies					
**Regimen**	Carboplatin Paclitaxel [32]	Carboplatin Paclitaxel Ramucirumab [34, 36]	Carboplatin Paclitaxel Atezolizumab [35]	Lenvatinib [43]	Pembrolizumab [44]	Lenvatinib Pembrolizumab [47]	Everolimus [58]	Palbociclib [59]	Sunitinib [42]
**Patients**	40	52	48	42	41	43	51	48	41
**ORR**	36%	80%	56%	38%	23%	23%	11%	13%	15%
**Median PFS (months)**	7.5	18.1	9.6	9.3	6.8	14.9	10.1	11.0	7.9
**PFSR-6 months**	65%	80%	75%	74%	50%	88%	52%	60%	58%

### Second-Line and Beyond Therapies

#### Cytotoxic Chemotherapy

In case of disease progression or unacceptable toxicity to first-line therapy, second-line chemotherapy options include carboplatin and paclitaxel or cisplatin and etoposide if not administered in the first line [6, 30]. Rechallenge of PAC is also to be considered in case of cumulative doses of anthracyclines have not been reached [6]. Capecitabine and gemcitabine, pemetrexed, oral etoposide, ifosfamide, paclitaxel are usually reserved for later lines [38, 39, 40, 41]. Response rates to second and further-line chemotherapy range from 15 to 40% [6, 30, 38, 39, 40, 41], with PFS around 6 months. No comparative studies have been conducted to identify the preferred treatment regimen (Table 1). Therefore, clinicians should base their choice on the previously administered agents and toxicity profile of each drug or combination. Participation in clinical trials is highly recommended.

#### Antiangiogenic Agents

A preferred option after first-line cytotoxic chemotherapy without antiangiogenic therapy may be to offer second-line therapy with single-agent antiangiogenic drugs including sunitinib or lenvatinib [42, 43]. In the landmark phase II trial with sunitinib, the ORR and disease control rate (DCR) in thymic carcinomas were 26% and 91%, and in thymomas 6% and 81% [48]; median PFS was about 7.2 and 8.5 months, respectively. Sunitinib may then represent an option as second-line treatment for any histology TETs. In the phase II REMORA trial, lenvatinib (at the dose of 24 mg) in thymic carcinoma patients [43] yelded aresponse rate of 38%, and median PFS and OS of9.3 months and 28.3 months, respectively. Therefore, lenvatinib may be considered as the standard option in patients with pre-treated metastatic thymic carcinoma. In thymic carcinomas and type B3 thymoma, after second-line treatment with one of these agents, rechallenge with chemotherapy or immunotherapy if not previously given are possible options.

#### Immune Checkpoint Inhibitors (ICIs)

ICIs such as pembrolizumab [44], nivolumab [45], and other agents, have been investigated in patients with advanced and previously-treated TETs, mostly thymic carcinomas and type B3 thymomas. These are contra-indicated in other subtype given a high risk of life-threatening irAEs, especially myocarditis [46]. Immunotherapy has demonstrated clinical activity, with reported ORR of 18–20% and 1-year PFS and OS rates of 25–30% and 60–70%, respectively [47]. As above mentioned, the intent of the treatment with ICIs relies on long-term response for a subset of patients which is yet to be demonstrated [46]. Even in thymic carcinoma, severe irAEs may occur in 25% of patients. Thus, close monitoring of patients is strongly advised [47]. For this reason, immunotherapy should be considered as third-line treatment after less risky regimens have been administered0.

Interestingly, some trials explored combinational approaches aiming to enhance immunotherapy efficacy. The CAVEATT trial reported data combining axitinib plus avelumab [48]; the PECATI trial investigated lenvatinib plus pembrolizumab with better outcomes than with each of these drugs and a median PFS of 14.9 months [49].

The role of potential biomarkers for immunotherapy efficacy remains controversial in TETs. PD-L1 expression is common in non-tumoral thymic epithelial cells and usually high and intense [50] which does not make PD-L1 a reliable criterion for treatment decisions. Tumor mutational burden (TMB) is uniformly low [51].

### Biomarker-Driven Targeted Therapies

Molecular alterations in oncogenic signaling pathways are observed in TETs. From The Cancer Genome Atlas and comprehensive genomic profiling data, the most frequent activating alterations predicting the efficacy of targeted agents are *KIT* (9% of cases, mainly in thymic carcinomas),*PI3K* mutations (5% of cases), and *CDKN2A/MTAP* loss (25–30% of cases) [51, 52]. Less frequent genomic alterations involve *BRAF*, *RET*, *NTRK*, *MET*, and *FGFR*. As targeted agents are in the clinic for these alterations, opportunities exist for precision medicine approaches in TETs, although limited evidence has been generated and the question on when these drugs should be implemented along the treatment sequence of TETs remains open.

#### KIT Mutations

When an activating *KIT* mutation is observed in TET, KIT inhibitors such as imatinib, sunitinib, or sorafenib, based on the sensitivity to these inhibitors reported in other cancers, like gastro-intestinal stromal tumors, may be considered [53, 54]. Response rates may be higher than 80%. However, no data are available on resistance mechanisms and sequences of KIT inhibitors.

#### Other Point Mutations

Anecdotal responses to targeted therapies in NTRK-positive or PI3K mutated TETs with specific inhibitors were reported [55, 56]. Discussion at a molecular multidisciplinary tumor board may facilitate the use of targeted agents [57].

### Non-Biomarker Driven Targeted Therapies

Everolimus was assessed in a phase II trial in advanced, refractory TETs [58, 59]. Stable disease was the most frequent response pattern, with DCR of 88% and ORRof 11%; median PFS was 10.1 months.

Palbociclib reported ORR of 13% and median PFS of 11.0 months; no selection was conducted based on CDK-related genes alterations.

Everolimus may represent an option in later lines, more for thymoma than thymic carcinomas, and in chemo-refractory tumors. Further evidence is probably required for palbociclib.

Octreotide has been historically used in advanced, refractory non-neuroendocrine TETs, with patients’ selection likely based on Octreoscan increased uptake, and in combination with steroids. Efficacy is unclear, and may be related to the lympholytic effect observed in type B thymomas with steroids [60].

Two phase II trials with PI3K inhibitors TAS-117 and buparlisib are currently recruiting patients with TETs. A pitfall of the latter trial is the absence of selection based on the identification of PI3K mutations.

### Treatment Sequences

All the above discussed treatment strategies, as well as next-generation-sequencing (NGS) molecular testing, have beenimplemented in the current management of patients with metastatic TETs. However, no phase III randomized trials have been conducted. Treatment choice, even when discussed at multidisciplinary tumor boards, still often relies on non-evidence-based expertise, and is therefore highly variable. To strengthen the decision-making and define the best treatment options and sequences, the following are the key questions for clinicians, especially for type B3 thymomas and thymic carcinomas, the most frequent subtypes associated with metastatic spread (Table 2):

1/ what is the best targeted agent after the failure of second-/third-linetherapy,

2/ is there any impact from biomarker selection on the efficacy of targeted agents in the second-/third-line setting, i.e. is there any predictive value of KIT, PI3K and CDKN2A mutations for the response to sunitinib, everolimus, and palbociclib, respectively, and which is the best of these three options in patients with no documented alterations.

3/ for type B3 thymoma and thymic carcinoma, what is the best treatment sequencing or combination option of chemotherapy, antiangiogenic agent and immunotherapy: chemotherapy alone followed by lenvatinib plus pembrolizumab (PECATI) or chemotherapy plus atezolizumab (MARBLE) followed by lenvatinib, or chemotherapy plus ramucirumab (RELEVENT) followed by immunotherapy, or even quadruplet with chemotherapy plus immunotherapy plus antiangiogenic upfront.

Whether novel strategies can be valuable, confirmation of their efficacy is needed in randomized studies given the heterogeneity of reported outcomes in single-arm trials. Considering patients for enrollment in clinical trials with novel agents is also a good practice in such rare cancers.

## Conclusion

The management of metastatic TETs represent a model for implementation and multimodal approaches within multidisciplinary teams. Over the past 5 years, the results of many trials have been reported enriching our treatment options (Figure), which, however, still need prioritization, optimization and correct sequencing. Networks of healthcare providers like the European Reference Network EURACAN dedicated to rare cancers are key in this matter; collaboration between experts centers allow regular virtual multidisciplinary tumor boards to be set up, real-world evidence to be generated, and collaborative research to be conducted. Such networkds represent a major tool to increased quality of care.

## Key References

- Vandaele T, Van Slambrouck J, Proesmans V, et al. Hyperthermic Intrathoracic Chemotherapy (HITHOC) for Pleural Disseminated Thymoma: A Systematic Literature Review. Ann Surg Oncol. 2023;30:543–560.
-
  - *Major review on HITHOC in thymic tumors*.
- Chun SG, Rimner A, Amini A, et al. American Radium Society Appropriate Use Criteria for Radiation Therapy in the Multidisciplinary Management of Thymic Carcinoma. JAMA Oncol 2023;9:971 − 80.
-
  - *American recommendations for radiotherapy in thymic tumors*.
- Petat A, Dansin E, Calcagno F, et al. Treatment strategies for thymic carcinoma in a real-life setting. Insights from the RYTHMIC network. Eur J Cancer. 2022;162:118–127.
-
  - *Large cohort of real-world data on systemic therapies for thymic carcinoma*.
- Proto C, Ganzinelli M, Manglaviti S, et al. Efficacy and safety of ramucirumab plus carboplatin and paclitaxel in untreated metastatic thymic carcinoma: RELEVENT phase II trial (NCT03921671). Ann Oncol. 2024;35:817–826.
-
  - *Results of the combination of chemotherapy and antiangiogenic in the first-line treatment of thymic carcinoma*.
- Mimori T, Shukuya T, Goto V, et al. Efficacy and safety of atezolizumab plus carboplatin and paclitaxel in patients with advanced or recurrent thymic carcinoma: MARBLE phase II study (JTD2101). ESMO Asia 2024.
-
  - *Results of the combination of chemotherapy and immunotherapy in the first-line treatment of thymic carcinoma*.
- Niho S, Sato J, Satouchi M, et al. Long-term follow-up and exploratory analysis of lenvatinib in patients with metastatic or recurrent thymic carcinoma: Results from the multicenter, phase 2 REMORA trial. Lung Cancer 2024;191:107557.
-
  - *Results of Lenvatinib as second-line and beyond treatment for thymic carcinoma*.
- Girard N, Basse C, Schrock A, et al. Comprehensive Genomic Profiling of 274 Thymic Epithelial Tumors Unveils Oncogenic Pathways and Predictive Biomarkers. Oncologist. 2022;27:919–929.
-
  - *Large cohort of comprehensive genomic profiling of thymic tumors*.
- Tagliamento M, Morfouace M, Loizides C, et al. EORTC-SPECTA Arcagen study, comprehensive genomic profiling and treatment adaptation of rare thoracic cancers. NPJ Precis Oncol. 2024;8:37.
-
  - *First report on molecular tumor board for thymic tumors*.

## Data Availability

No datasets were generated or analysed during the current study.
